# Coexisting focal nodular hyperplasia and diffuse hepatic metastases from an ileal neuroendocrine tumor: a case report and literature review

**DOI:** 10.1097/RC9.0000000000000978

**Published:** 2026-09-14

**Authors:** Olfa Ghannei, Hamza Sboui, Mayada Ben Trimech, Soumaya Ben Amor

**Affiliations:** Department of Gastroenterology, University Hospital of Mahdia, Mahdia, Tunisia

**Keywords:** focal nodular hyperplasia, hepatic imaging, hepatic metastases, neuroendocrine tumor

## Abstract

**Introduction and importance:**

Focal nodular hyperplasia (FNH) is a benign liver lesion usually associated with vascular abnormalities. Neuroendocrine tumors (NETs), on the other hand, frequently metastasize to the liver and typically appear as hypervascular lesions. The coexistence of FNH and diffuse hepatic metastases from an ileal NET is exceptionally rare and represents a major diagnostic challenge because both entities may present as hypervascular liver lesions, potentially leading to misdiagnosis and inappropriate management.

**Case presentation:**

A 56-year-old woman with a history of epilepsy treated with sodium valproate presented with progressive right upper quadrant pain and abdominal distension. Imaging showed a large hepatic lesion suggestive of FNH along with multiple hypervascular liver nodules suspicious for metastases. A CT-guided liver biopsy demonstrated metastatic well-differentiated NET with a Ki-67 index below 2%. Laboratory tests revealed elevated chromogranin A and urinary 5-hydroxyindoleacetic acid levels. The patient also had chronic diarrhea and moderate tricuspid regurgitation consistent with carcinoid syndrome. A ^68 Ga-DOTANOC positron emission tomography/computed tomography localized the primary tumor to the ileum. The integration of radiological, functional, and histopathological findings enabled accurate diagnosis and appropriate therapeutic planning. Surgical resection of the primary tumor was performed, followed by long-acting octreotide therapy. The patient remained clinically stable, with no progression of the hepatic lesions during follow-up.

**Clinical discussion:**

The presence of multiple hypervascular liver lesions in patients with NETs raises important diagnostic issues, especially when benign and malignant lesions coexist. This case demonstrates the value of integrating hepatobiliary magnetic resonance imaging (MRI), histopathological confirmation, and functional imaging to accurately distinguish FNH from metastatic disease and to guide appropriate therapeutic management.

**Conclusion:**

The coexistence of FNH and diffuse liver metastases from an ileal NET is rare. This case highlights the pivotal role of a multimodal diagnostic approach combining hepatobiliary MRI, functional imaging, and targeted histopathological confirmation to distinguish benign from malignant hypervascular liver lesions, avoid diagnostic pitfalls, and optimize patient management.

## Introduction

Neuroendocrine tumors (NETs) are uncommon neoplasms characterized by frequent hepatic dissemination, particularly in small-bowel primaries. Liver metastases are present in up to 75% of patients with advanced disease and largely determine prognosis and therapeutic management^[^1^]^. Owing to their marked arterial hypervascularity, NET metastases may mimic several benign and malignant hepatic lesions on imaging studies, thereby creating important diagnostic challenges.

Focal nodular hyperplasia (FNH) is the second most common benign hepatic lesion after hemangioma and is generally considered a regenerative hyperplastic response to localized vascular abnormalities. Typical imaging findings on contrast-enhanced magnetic resonance imaging (MRI), particularly with hepatobiliary contrast agents, usually allow a confident non-invasive diagnosis^[^2^]^. However, atypical presentations or coexistence with malignant hypervascular lesions may complicate radiologic interpretation.

The synchronous coexistence of FNH and neuroendocrine liver metastases is exceptionally rare and may lead to diagnostic confusion, particularly when multiple hypervascular hepatic lesions are present. Distinguishing benign from metastatic lesions is crucial, as management strategies and prognostic implications differ substantially. We report a rare case of a large FNH coexisting with diffuse hepatic metastases from a grade 1 ileal NET, highlighting the importance of multimodal imaging, histopathological confirmation, and multidisciplinary evaluation. This case report was prepared in accordance with the SCARE checklist^[^3^]^.

## Case presentation

A 56-year-old woman with a long-standing history of epilepsy treated with sodium valproate since the age of 19 presented with atypical pain in the right upper quadrant, associated with progressive abdominal distension. She reported recurring episodes of diarrhea over the past 3 months. She denied weight loss or fever.

Physical examination revealed a body mass index of 22 kg/m^2^. Abdominal examination revealed a firm mass measuring approximately 9 cm that was palpable in the right hypochondrium and extended into the epigastrium. There were no signs of portal hypertension, such as splenomegaly or ascites, and no peripheral lymphadenopathy.

Laboratory tests revealed mild hepatic cytolysis with alanine aminotransferase of 60 IU/L and aspartate aminotransferase of 80 IU/L. Gamma-glutamyl transferase was 34 IU/L, and alkaline phosphatase was 150 IU/L. Total bilirubin was 16 µmol/L. Liver synthetic function was preserved, with a prothrombin time of 100% and serum albumin of 41 g/L. Viral hepatitis serologies were negative, and there was no history of chronic liver disease, alcohol misuse, or oral contraceptive exposure. **Tumor markers**, including alpha-fetoprotein, carcinoembryonic antigen, and carbohydrate antigen 19–9, were within the normal range.

Abdominal ultrasound demonstrated a large hepatic mass arising from the left lobe measuring approximately 9 cm, associated with multiple nodular lesions scattered throughout the liver. Contrast-enhanced computed tomography and liver MRI were subsequently performed. On MRI, the dominant lesion appeared iso- to hypointense on T1-weighted images and slightly hyperintense on T2-weighted sequences, with a central stellate scar showing delayed enhancement. Diffusion restriction was absent, with an apparent diffusion coefficient (ADC) of 1.8 × 10^−3^ mm^2^/s. Following administration of gadoxetate disodium (Gd-EOB-DTPA, Primovist^®^, Bayer), the lesion demonstrated intense homogeneous arterial enhancement with persistent hepatobiliary phase enhancement, strongly suggestive of FNH (Fig. 1). Given the highly characteristic MRI appearance, biopsy of the dominant lesion was not considered necessary.

**Figure 1.**
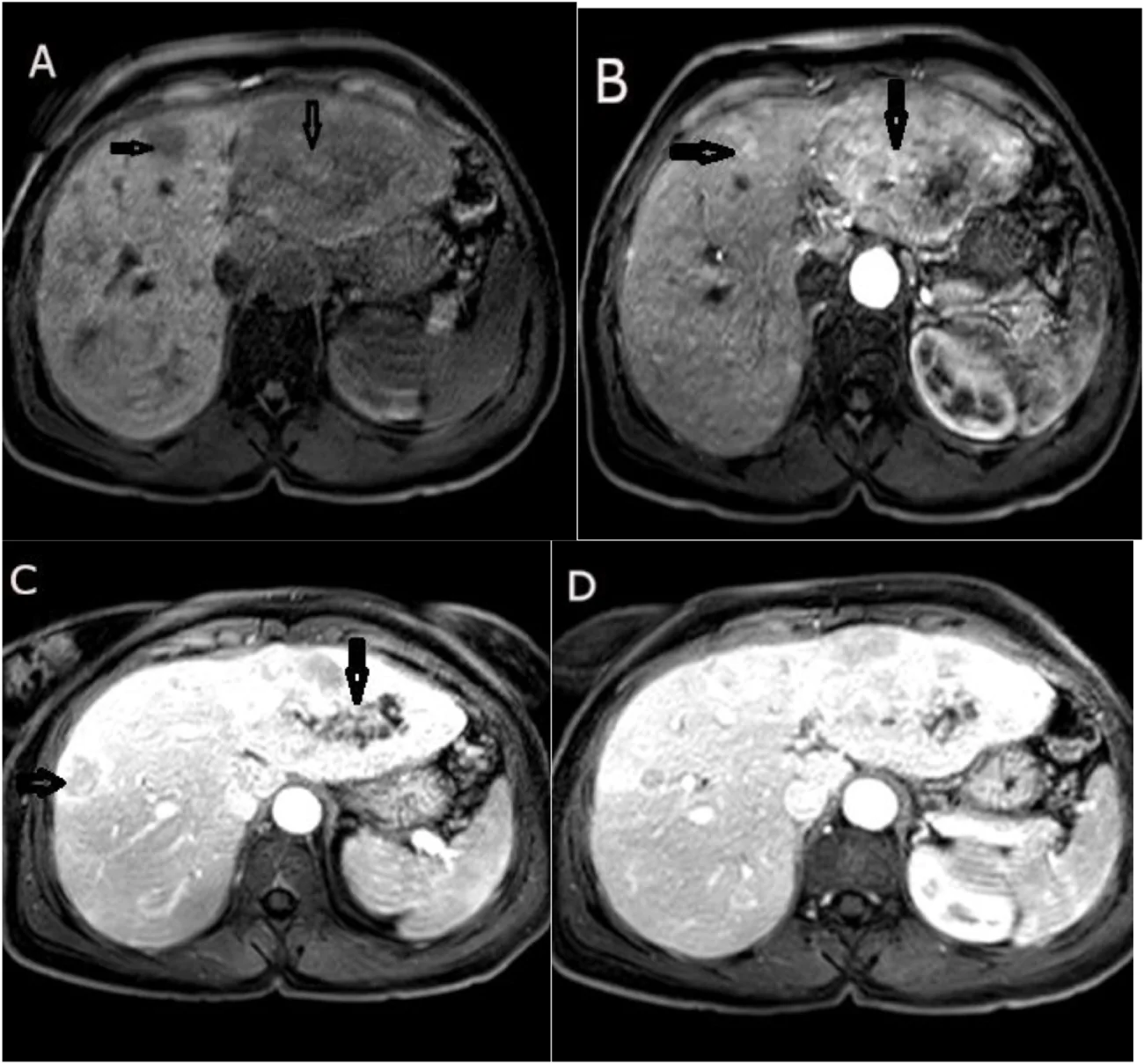
Multiphasic liver MRI demonstrating focal nodular hyperplasia coexisting with diffuse neuroendocrine liver metastases. (A) Axial pre-contrast T1-weighted MRI showing a large left hepatic lobe lesion associated with multiple smaller hepatic nodules. (B) Arterial phase imaging demonstrating intense homogeneous arterial enhancement of the dominant lesion, suggestive of focal nodular hyperplasia. (C) Hepatobiliary phase imaging showing persistent contrast uptake within the dominant lesion, consistent with preserved hepatocellular function. (D) The smaller metastatic nodules remain hypointense during the hepatobiliary phase, consistent with neuroendocrine liver metastases lacking functioning hepatocytes.

In contrast, numerous hypervascular nodules measuring between 5 and 28 mm were identified throughout both hepatic lobes, raising suspicion for metastatic disease. These lesions demonstrated diffusion restriction with an ADC of 0.5 × 10^−3^ mm^2^/s. Further investigations, including upper gastrointestinal endoscopy and colonoscopy, did not reveal any lesions.

CT-guided biopsy of one of these nodules confirmed metastatic well-differentiated NET. Immunohistochemical analysis showed strong positivity for chromogranin A and synaptophysin, with a Ki-67 proliferation index below 2%, consistent with grade 1 NET (Fig. 2). Biochemical evaluation demonstrated elevated serum chromogranin A and increased urinary 5-hydroxyindoleacetic acid levels. Clinically, the patient reported chronic watery diarrhea, and transthoracic echocardiography demonstrated moderate tricuspid regurgitation, compatible with carcinoid heart disease. No other significant echocardiographic abnormalities were identified.

**Figure 2.**
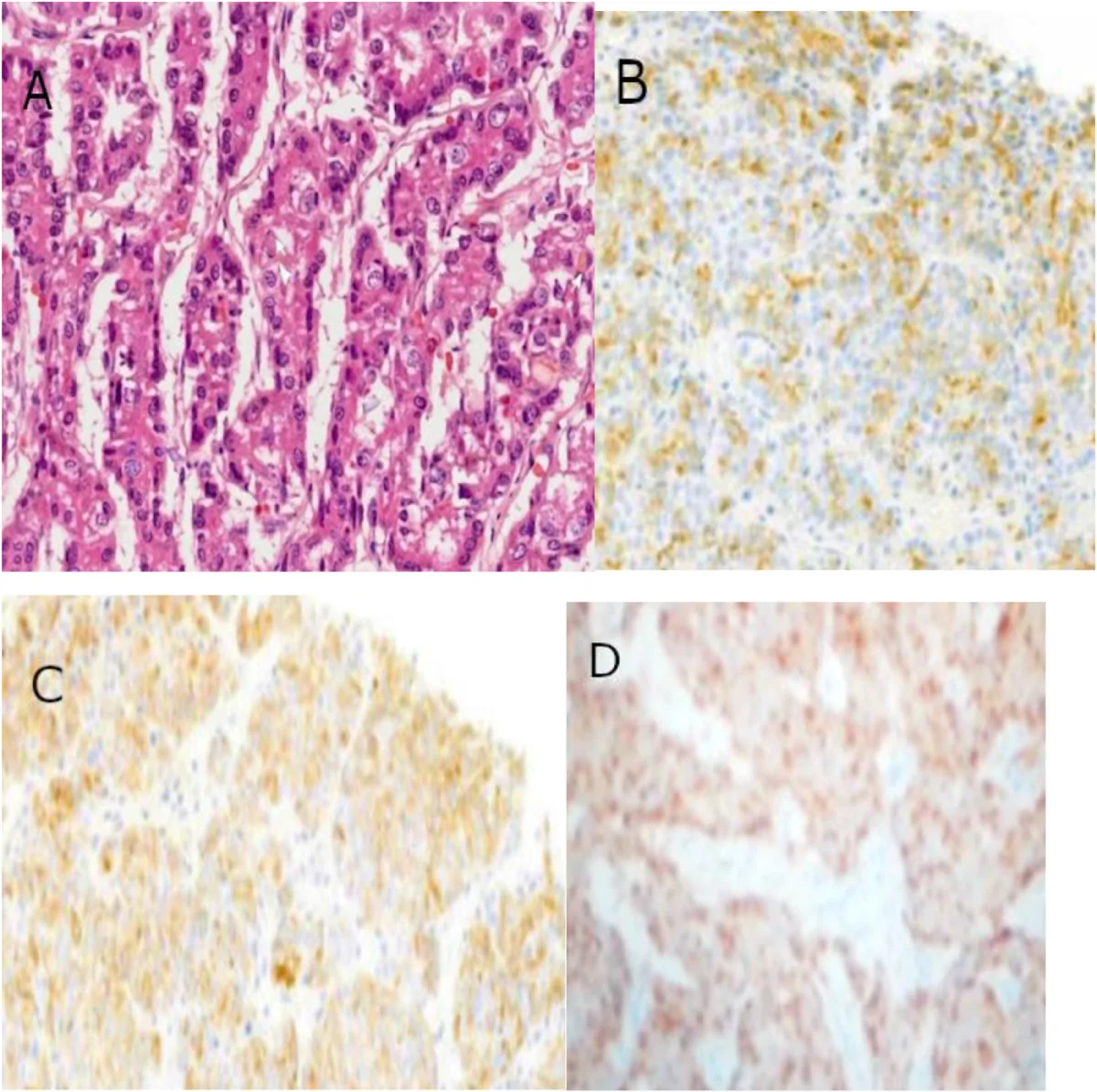
Microscopic and immunophenotypic findings from the liver biopsy confirm a neuroendocrine neoplasm. (A) Hematoxylin and eosin (H&E) staining showing organoid and nested patterns of uniform tumor cells. (B) Strong and diffuse cytoplasmic positivity for Chromogranin A. (C) Immunohistochemical staining demonstrating membranous and cytoplasmic expression of Synaptophysin. (D) Ki-67 proliferation index demonstrating nuclear positivity in tumor cells.

A 68 Ga-DOTANOC positron emission tomography/computed tomography (PET/CT) scan identified the primary lesion in the ileum. The metastatic hepatic lesions demonstrated intense somatostatin receptor expression on ^68 Ga-DOTANOC PET/CT (SUVmax 29.3), whereas the dominant hepatic lesion showed only minimal tracer uptake (SUVmax 3.2), further supporting its benign nature. The patient underwent minimally invasive surgical management by laparoscopic segmental ileal resection. Exploration confirmed a localized ileal tumor measuring approximately 28 mm in diameter, associated with multiple enlarged mesenteric lymph nodes, without evidence of peritoneal carcinomatosis. A segmental ileal resection with adequate proximal and distal margins was performed together with regional mesenteric lymphadenectomy, preserving the main mesenteric vascular supply. Intestinal continuity was restored using a side-to-side stapled entero-enteric anastomosis. The postoperative course was uneventful. Histopathological examination confirmed a well-differentiated, grade 1, ileal NET (Ki-67 < 2%). Metastatic involvement was identified in 2 of the 12 resected mesenteric lymph nodes (2/12). According to the AJCC 8th edition TNM classification, the tumor was staged as pT3N1M1, G1 (Stage IV). Postoperative recovery was uncomplicated.

HIGHLIGHTS

- Coexistence of focal nodular hyperplasia and diffuse hepatic metastases from an ileal neuroendocrine tumor is exceptionally rare.
- Benign and malignant hypervascular liver lesions may coexist, creating a major diagnostic pitfall.
- Hepatobiliary magnetic resonance imaging, targeted liver biopsy, and ^68 Ga-DOTANOC positron emission tomography/computed tomography were complementary for accurate diagnosis.
- Targeted histopathological confirmation of suspicious lesions is essential when imaging findings are atypical or discordant.
- A multidisciplinary approach is crucial for appropriate therapeutic decision-making.

Following surgery, first-line treatment with long-acting octreotide was initiated. The patient received octreotide LAR 30 mg administered intramuscularly every 4 weeks. Treatment was well tolerated without adverse events requiring dose modification. At 6-month follow-up, the patient remained clinically stable. Diarrhea markedly improved, abdominal discomfort resolved, and no new carcinoid-related symptoms developed. Follow-up contrast-enhanced CT imaging demonstrated stable hepatic metastatic disease according to RECIST criteria, with no evidence of new lesions or progression. The dominant hepatic lesion remained radiologically stable, measuring 8.9 cm compared with approximately 9.0 cm at baseline. The patient continues regular multidisciplinary follow-up while receiving monthly octreotide therapy. Given the lesion’s large size and the lack of histopathological confirmation of the dominant hepatic lesion, liver MRI surveillance at 6-month intervals has been planned to monitor its size and imaging characteristics.

## Discussion

NETs are relatively uncommon neoplasms that most frequently arise within the gastrointestinal tract, particularly the small intestine and pancreas. Despite their typically indolent growth pattern, these tumors have a strong tendency for hepatic dissemination, with liver metastases occurring in approximately 50–75% of patients with advanced disease. Hepatic involvement plays a major role in prognosis and is frequently responsible for hormonal manifestations, such as carcinoid syndrome^[^4^]^.

Radiologically, hepatic NET metastases classically present as hypervascular lesions demonstrating intense arterial phase enhancement, occasionally associated with portal or delayed phase washout. However, these enhancement patterns may overlap with those of benign hypervascular hepatic lesions, particularly FNH and hepatocellular adenoma, thereby creating significant diagnostic uncertainty. Inflammatory hepatocellular adenoma deserves particular consideration because it may occasionally demonstrate hepatobiliary phase uptake and mimic FNH on MRI. Nevertheless, the presence of a central stellate scar, a homogeneous enhancement pattern, and persistent imaging stability in our patient favored a diagnosis of FNH.

FNH is the second most common benign hepatic lesion after hemangioma and predominantly affects women of reproductive age^[^5^]^. It is considered a localized hyperplastic response to an underlying vascular malformation and is usually discovered incidentally in asymptomatic patients. FNH carries no recognized risk of malignant transformation or significant hemorrhage and generally follows a benign clinical course. Consequently, conservative management is recommended for asymptomatic patients with a confident diagnosis, whereas surgery is reserved for symptomatic cases or persistent diagnostic uncertainty. Although spontaneous rupture has been reported in exceptional cases, close radiological follow-up is generally preferred, particularly for lesions larger than 5 cm^[^6^]^.

An additional point of interest is the patient’s 37-year history of sodium valproate therapy. Although valproate is primarily associated with hepatotoxicity, isolated reports have suggested a possible link to FNH-like lesions and hepatic vascular alterations. While no causal relationship can be established, long-term valproate exposure may have contributed to the vascular changes underlying FNH in this patient.

MRI using hepatocyte-specific contrast agents currently represents the most reliable non-invasive imaging modality for the diagnosis of FNH. Typical imaging features include intense homogeneous arterial enhancement, a central stellate scar, absence of significant diffusion restriction, and persistent enhancement during the hepatobiliary phase, reflecting preserved hepatocellular function within the lesion. When these highly characteristic imaging features are present, the diagnosis of FNH can usually be established with high confidence, thereby avoiding unnecessary biopsy or surgical intervention^[^7^]^.

However, significant diagnostic challenges may arise when FNH coexists with other hypervascular hepatic lesions, particularly in oncologic settings. Previous studies have described synchronous associations between FNH and hepatocellular adenoma or hepatocellular carcinoma, emphasizing that lesions with distinct histopathological origins may coexist within the same liver^[^5,6,8^]^. In such situations, radiological interpretation may become particularly challenging because some malignant lesions, especially hypervascular metastases, can partially mimic benign enhancement patterns.

This diagnostic dilemma is especially relevant in NETs, whose liver metastases frequently demonstrate marked arterial hyperenhancement. Although NET metastases typically appear hypointense during the hepatobiliary phase owing to the absence of functioning hepatocytes, atypical hepatobiliary phase enhancement has occasionally been reported, resulting in potential diagnostic overlap with benign hypervascular lesions^[^9^]^. In rare instances, metastatic lesions with neuroendocrine differentiation have shown imaging features resembling FNH on contrast-enhanced studies, ultimately requiring histopathological confirmation for definitive diagnosis.

In the present case, the dominant hepatic lesion demonstrated highly characteristic imaging features of FNH, including homogeneous arterial enhancement, a central stellate scar, persistent hepatobiliary phase uptake, absence of diffusion restriction (ADC 1.8 × 10^−3^ mm^2^/s), minimal somatostatin receptor expression (SUVmax 3.2), and radiological stability during follow-up. In contrast, the smaller hepatic nodules demonstrated diffusion restriction (ADC 0.5 × 10^−3^ mm^2^/s) and intense somatostatin receptor expression on ^68 Ga-DOTANOC PET/CT (SUVmax 29.3), findings consistent with metastatic disease. Therefore, although atypical hypervascular NET metastases may occasionally resemble FNH on hepatobiliary MRI, this possibility was considered unlikely in our patient. Histopathological analysis obtained through CT-guided biopsy confirmed a well-differentiated metastatic NET characterized by positive chromogranin A and synaptophysin expression and a low Ki-67 proliferation index (<2%), consistent with a grade 1 tumor.

Only a few cases describing the association between FNH and neuroendocrine neoplasms have been reported in the literature (Table 1). Most involved diagnostic confusion between benign hypervascular lesions and metastatic disease rather than true synchronous coexistence. Compared with these reports, our case is distinguished by the simultaneous presence of a giant typical FNH and diffuse bilobar liver metastases from a grade 1 ileal NET at initial presentation. The patient’s right upper quadrant pain was most likely attributable to the giant presumed FNH because of its large size and subcapsular location, whereas well-differentiated liver metastases are frequently asymptomatic unless associated with extensive tumor burden. Although the diagnostic challenge was similar, the coexistence of benign and malignant lesions in our patient required a multimodal diagnostic approach combining hepatobiliary MRI, targeted biopsy of suspicious lesions, and ^68 Ga-DOTANOC PET/CT to accurately characterize the hepatic lesions and guide appropriate management. This case, therefore, expands the limited literature on this rare association and highlights the importance of integrating radiological, functional, and histopathological findings to avoid both overtreatment of benign lesions and underestimation of metastatic disease.

**Table 1:** Reported cases of focal nodular hyperplasia associated with neuroendocrine tumors and related diagnostic challenges.

Ref.	Sex/age	Pathology – size (cm)	Location (segment)	Symptoms		Treatment	Diagnostic pitfall	Histology
Jung *et al*, 2015^[^10^]^	Female, child	Multifocal FNH developing after resection of pancreatic insulinoma (NET); multiple nodules	Multiple hepatic segments	Asymptomatic lesions discovered during postoperative follow-up imaging		Surgical resection of insulinoma; conservative follow-up for FNH	Suspected liver metastases	Confirmed
Gainey *et al*, 2020^[^11^]^	Male, adult	FNH occurring after liver transplantation in a patient with prior carcinoid tumor; solitary hypervascular lesion	Liver graft (segment not specified)	Incidental hypervascular mass suspicious for recurrence		Histological confirmation; conservative management	Suspected recurrence	Confirmed
Present case	Female, adult	Giant focal nodular hyperplasia (9 cm) associated with multifocal bilobar hepatic metastases from grade 1 ileal neuroendocrine tumor (Ki-67 <2%)	Dominant lesion arising from the left hepatic lobe with multiple bilateral metastatic nodules	Right upper quadrant pain, abdominal distension, chronic watery diarrhea, carcinoid syndrome with tricuspid insufficiency	Surgical resection of primary ileal NET and treatment with long-acting somatostatin analog (octreotide LAR)		FNH + NET metastases coexistence	NET biopsy confirmed

Beyond conventional imaging, functional imaging also plays a pivotal role in the diagnostic work-up. Functional imaging based on somatostatin receptor expression has become an essential component in the evaluation of NETs. In the present case, ^68 Ga-DOTANOC PET/CT successfully localized the primary ileal tumor and accurately assessed the extent of hepatic metastatic involvement. Compared with conventional imaging modalities, somatostatin receptor PET/CT demonstrates high sensitivity for detecting well-differentiated NETs and currently represents a key imaging modality for staging and therapeutic decision-making^[^12^]^. In our patient, quantitative imaging findings further strengthened the diagnostic confidence, as the dominant hepatic lesion demonstrated minimal somatostatin receptor uptake and no diffusion restriction, whereas the metastatic lesions showed intense tracer uptake and marked diffusion restriction, supporting the distinction between the dominant hepatic lesion, presumed to represent FNH, and neuroendocrine liver metastases. This case emphasizes that accurate multimodal characterization of hepatic lesions is essential to avoid inappropriate therapeutic decisions in patients with NETs.

The therapeutic management of metastatic NETs is guided by tumor differentiation, Ki-67 proliferative index, somatostatin receptor expression, symptom burden, and extent of metastatic spread. In our patient, the presence of diffuse bilobar type III neuroendocrine liver metastases, according to the modified Frilling classification^[^13^]^, precluded curative hepatic resection or local ablative therapies. Given the diffuse bilobar metastatic distribution and the presumed benign nature of the dominant FNH lesion, combined bowel resection and major hepatectomy would not have provided a favorable risk-benefit balance. Given the low Ki-67 proliferative index, positive somatostatin receptor imaging, and relatively indolent tumor behavior, first-line treatment with long-acting octreotide was initiated in accordance with current recommendations for well-differentiated metastatic NETs. In patients with diffuse bilobar neuroendocrine liver metastases, treatment should be individualized within a multidisciplinary setting. Liver-directed therapies, including cytoreductive surgery or embolization, are generally reserved for selected patients with limited or liver-dominant disease, whereas peptide receptor radionuclide therapy is generally considered for patients with progressive, somatostatin receptor-positive disease despite somatostatin analog therapy. Resection of the primary ileal tumor was nevertheless performed to reduce the risk of future intestinal and mesenteric complications and to improve hormonal symptom control. Under somatostatin analog therapy, the patient demonstrated stable hepatic disease at 6-month follow-up^[^14^]^.

## Strengths and limitations

This case report has several strengths. It describes an exceptionally rare synchronous coexistence of FNH and diffuse liver metastases from an ileal NET. The diagnosis was supported by a multimodal approach integrating hepatobiliary MRI, targeted histopathological examination of the metastatic lesions, and ^68 Ga-DOTANOC PET/CT, complemented by a focused review of the available literature. One important limitation of this case is the absence of histopathological confirmation of the dominant hepatic lesion presumed to represent FNH. Although biopsy remains the reference standard, it was intentionally avoided because the lesion exhibited highly characteristic MRI features – including homogeneous arterial hyperenhancement, a central stellate scar, persistent hepatobiliary phase enhancement, and absence of diffusion restriction – and remained radiologically stable during follow-up. This approach is consistent with current EASL Clinical Practice Guidelines, which do not recommend biopsy for lesions demonstrating typical imaging features of FNH on hepatobiliary MRI unless diagnostic uncertainty persists. Nevertheless, we acknowledge that the diagnosis ultimately relied on imaging criteria rather than histological confirmation, which should be considered when interpreting this case.

Another limitation is that only one hepatic metastasis was biopsied, preventing assessment of potential intrapatient tumor heterogeneity.

Moreover, the relatively short 6-month follow-up limits the evaluation of the long-term stability of the presumed FNH lesion and the evolution of metastatic disease. Given the large size of the dominant lesion and the absence of histopathological confirmation, continued radiological surveillance has been planned, with liver MRI at 6-month intervals to monitor lesion size and imaging characteristics. Any increase in size or change in its previously characteristic imaging features would prompt multidisciplinary reassessment and consideration of histopathological confirmation.

## Conclusion

This case highlights the rare coexistence of FNH and diffuse hepatic metastases from a well-differentiated ileal NET. Because both entities may demonstrate hypervascular enhancement patterns, significant diagnostic confusion can occur.

Although multiparametric MRI can confidently diagnose typical FNH, histopathological confirmation should still be considered when imaging findings are atypical or when benign and malignant hypervascular lesions coexist. In our case, the absence of histopathological confirmation of the dominant FNH lesion is an acknowledged limitation.

## Data Availability

All data generated or analyzed during this study are included in this published article.
